# LC-ESI-MS/MS Analysis and Pharmacokinetics of Plantainoside D Isolated from *Chirita longgangensis* var. *hongyao*, a Potential Anti-Hypertensive Active Component in Rats

**DOI:** 10.3390/molecules190915103

**Published:** 2014-09-22

**Authors:** Manyuan Wang, Shujun Fu, Xinshi Zhang, Jing Li, Muxin Gong, Feng Qiu

**Affiliations:** 1 Beijing Key Lab of TCM Collateral Disease Theory Research, School of Traditional Chinese Medicine, Capital Medical University, Beijing 100069, China; E-Mails: wangmyjun@aliyun.com (M.W.); liyangziyi103@hotmail.com (J.L.); gongmuxin@126.com (M.G.); 2 Faculty of Chinese Materia Medica, Tianjin University of Traditional Chinese Medicine, Tianjin 300193, China; E-Mail: fushujun90@aliyun.com; 3 Department of Pharmacy, Hebei North University, Zhangjiakou 075000, Hebei, China; E-Mail: zhangxinshi113@163.com

**Keywords:** *Chirita longgangensis* var. *hongyao*, plantainoside D, bioavailability, LC-ESI-MS/MS, rat

## Abstract

Plantainoside D (PD) is a potential anti-hypertensive active ingredient newly isolated from the dried plants of *Chirita longgangensis* var. *hongyao*. A sensitive and specific LC-ESI-MS/MS method was first developed and validated for the analysis of PD in rat plasma using genistein as the internal standard (IS). The plasma samples were pretreated with methanol-acetonitrile (50:50, v/v) to precipitate protein, and then chromatographed on a reverse-phase Agilent Zorbax XDB C18 column (50 mm × 2.1 mm, 3.5 μm). Gradient elution was utilized, with a mobile phase consisting of water and acetonitrile both containing 0.1% formic acid, and the flow rate was set at 0.50 mL/min. The analytes were monitored by tandem-mass spectrometry with negative electrospray ionization. The precursor/product transitions (*m/z*) in the negative ion mode were 639.2 → 160.9 Thomson (Th) and 268.9 → 158.9 Thomson (Th) for PD and IS, respectively. Linearity was achieved in the 0.10–200 ng/mL range, with a lower limit of quantification of 0.10 ng/mL. The precision and accuracy for both intra- and inter-day determination of the analyte were all within ±15%. The present method has been applied for pharmacokinetic study of PD after oral and intravenous administration in rats. The oral absolute bioavailability (F) of PD in rats was estimated to be 1.12% ± 0.46% with an elimination half-life (t_1/2_) value of 1.63 ± 0.19 h, suggesting its poor absorption and/or strong metabolism *in vivo*.

## 1. Introduction

**Figure 1:**
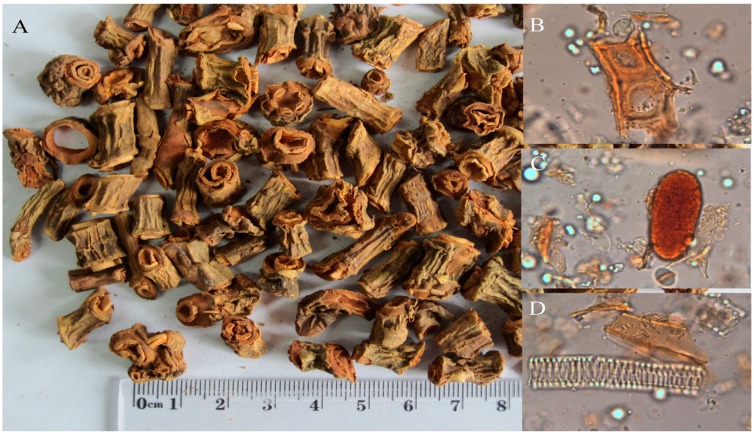
Pictures of *C. longgangensis* var. *hongyao* medicinal material and its characteristic micrographs. (**A**) *C. longgangensis* var. *hongyao* medicinal material; (**B**) sclerenchymatous cell; (**C**) eleocyte; (**D**) reticulate vessel.

*Chirita longgangensis* W. T. Wang var. *hongyao* S. Z. Huangisa popularly used folk herbal medicine named “Hongyao”, found distributed only in the south of China, especially in Guangxi Province [1]. *C. longgangensis* var. *hongyao* has the functions of promoting the formation of hemoglobin and red blood cell, and easing pain. Thus, it has long been used as a folk medicine in China forthe treatment of arthritis, anemia and fractures [1,2]. Figure 1 shows pictures of *C. longgangensis* var. *hongyao* medicinal material and its characteristic micrographs. It can be used for the treatment of the women’s diseases and also be used as an analgesic and antioncotic crude drug [2].

**Figure 2:**
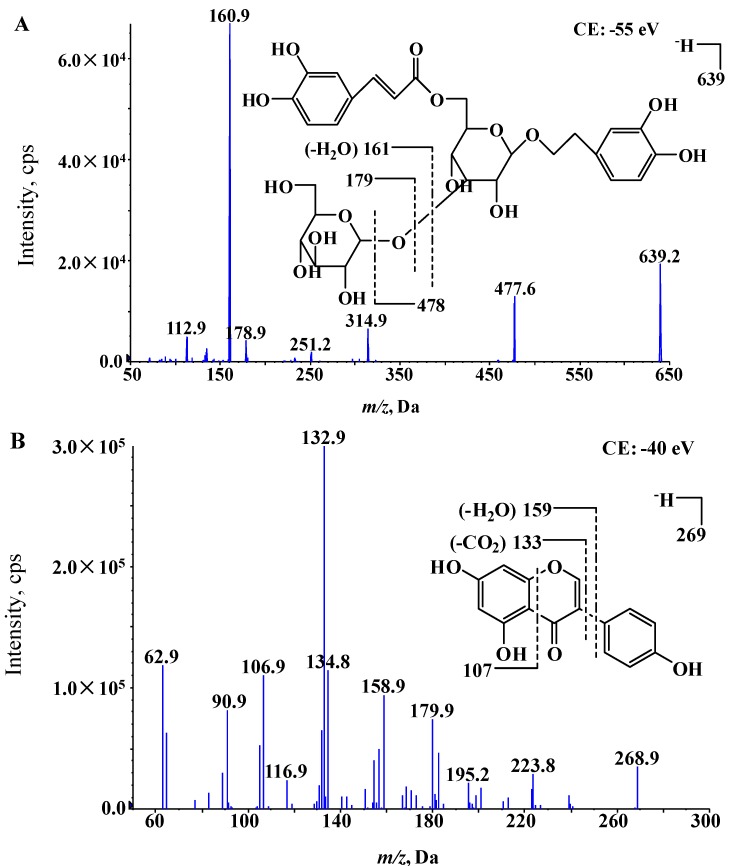
Full-scan product ion spectra of [M−H]^−^ ions and fragmentation schemes for (**A**) PD and (**B**) genistein (internal standard).

In recent years, preliminary phytochemical studies have been performed in our laboratory to isolate and determine the flavonoids and phenylethanoid glycosides from *C. longgangensis* var. *hongyao* [3,4]. Plantainoside D (PD, Figure 2), a major pharmacological active ingredient, is one of the most abundant components in *C. longgangensis* var. *hongyao* and other similar plants [5,6,7,8,9].

Preliminary pharmacological studies have demonstrated that PD showed strong angiotensin-converting enzyme inhibitive effects, radical scavenging activity and antioxidant effects [10,11,12,13,14]. Significant inhibitory activity of angiotensin-converting enzyme (ACE) of PD was confirmed by monitoring *in vitro* the transformation of a hippuryl-histidyl-leucine (HHL) substrate into the product hippuric acid (HA). The IC_50_ value of PD was estimated to be 2.17 mM [10]. PD can inhibit adriamycin (ADR)-induced apoptosis in H9C2 cardiac muscle cells via inhibition of ROS generation and NF-kappa B activation [11]. Therefore, PD is now being considered as a candidate drug using for cardiotoxic protection for ADR-exposed patients.

A RP-HPLC method has been developed in our laboratory for simultaneous determination of plantainoside D and verbascoside from the stems of *C. longgangensis* var. *hongyao* [4]. However, this quality control method for the medicinal material requires an efficient clean-up procedure, shorter running time and higher sensitivity. Till now, there are no reported bioanalytical methods for the quantification of PD in biological fluids and thus its pharmacokinetic profile has not been investigated. In present paper, we focused on developing a sensitive and specific LC-ESI-MS/MS method for the determination of PD in rat plasma. The method has then been successfully applied to the pharmacokinetic evaluation of PD using the rat as an animal model.

## 2. Results and Discussion

### 2.1. Mass Spectrometry and Chromatography

First a sensitive and specific LC-MS/MS assay has been developed for the determination of PD in rat plasma. The full-scan product ion mass spectra and fragmentation schemes of PD and genistein (internal standard) are shown in Figure 2. In the full-scan Q1 mass spectrum, the parent negative ion peak of PD appeared at *m/z* 639.2, and the abundance of this ion peak was sufficient for the quantification of PD. The most abundant product ion (*m/z* 160.9) was chosen to be the quantifying product ion, and it was inferred to correspond to the fragmentation of glucose from the whole molecule and consecutive loss of a water molecule. In addition, a second product ion *m/z* 477.6 was also employed as an additional product ion for further confirmation. For genistein, the most abundant peak was the protonated molecular ion [M−H]^−^ found at *m/z* 268.9. Two most abundant product ions of genistein (*m/z* 158.9 and *m/z* 132.9) were compared to confirm their suitability to be the quantifying product ion. It was found that the signal response from the *m/z* 132.9 product ion was not stable enough to be employed. Thus the mass transition was monitored for *m/z* 268.9 → 158.9. Finally, deprotonated PD and genistein were targeted for fragmentation, and the most stable and abundant ions in the product ion scan of PD and genistein were *m/z* 160.9 and 158.9, respectively. Subsequently, the mass transitions were monitored at *m/z* 639.2 → 160.9 for PD and *m/z* 268.9 → 158.9 for genistein. Other conditions such as ion spray voltage, curtain gas pressure, nebulizer gas pressure, heater gas pressure, source temperature and collision energy were further optimized to improve the PD sensitivity and response stability.

**Figure 3:**
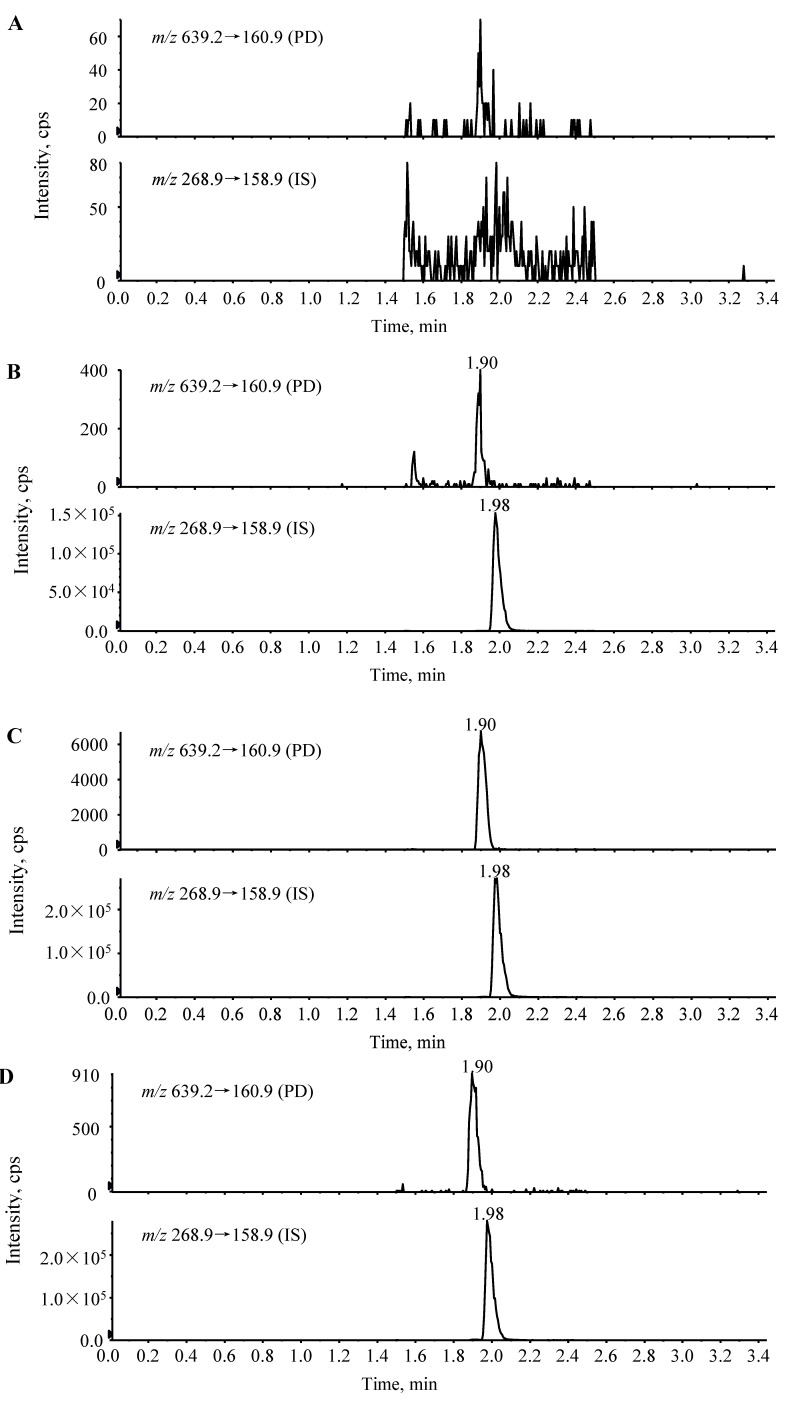
Typical chromatograms of (**A**) blank rat plasma; (**B**) blank rat plasma spiked with PD (0.1 ng/mL, LLOQ) and IS; (**C**) an unknown rat plasma sample collected at 15 min after intravenous administration of 2.0 mg/kg PD; and (**D**) an unknown rat plasma sample collected at 30 min after oral administration of 10 mg/kg PD.

LC-MS/MS chromatograms of PD and IS obtained by pretreatment of blank rat plasma, blank plasma spiked with PD and IS, and actual unknown plasma samples obtained in rats after intravenous and oral injection of PD are shown in Figure 3. The chromatographic run time for the extracted plasma samples was 3.5 min. The retention times for PD and IS were 1.90 and 1.98 min, respectively. The chromatograms show baseline separation of PD and the internal standard without any interference from endogenous plasma components.

During the optimization of chromatographic conditions, PD was extensively retained on several kinds of columns due to its strong lipophilicity. To achieve symmetric peak shapes and short chromatographic running times, the mobile phase consisting of acetonitrile with 0.1% formic acid and water with 0.1% formic acid was used on a Zorbax XDB C_18_ column.

An internal standard is usually required in LC-MS/MS analysis in order to rectify the probable errors in sample processing and determination. Usually an isotopically labeled internal standard (in this case, deuterated PD) would be an ideal choice, however, it was not available during the method development period, so in this study, genistein, a readily available natural compound which displays similar chromatographic retention behavior (t_R_= 1.98 min) as PD and high extraction efficiency (>80%) was selected as the IS. In addition, there were no interferences on the IS from PD or endogenous substances.

### 2.2. Linearity, Sensitivity and Detection Limit of the Assay

Calibration standards were prepared by spiking 5 μL of the appropriate standard solutions of PD into 50 μL of blank rat plasma. Plasma concentrations were 0.10, 0.20, 0.50, 1.0, 2.0, 10, 50, 100 and 200 ng/mL for PD. The peak area (*y*) and concentration of PD (*x*) were subjected to a weighted (1/X^2^) least squares linear regression analysis to calculate calibration equation and correlation coefficients. The linear ranges of PD in rat plasma were from 0.10–200 ng/mL. The lower limit of quantification (LLOQ) of PD was 0.10 ng/mL. Typical equations for the standard curves were *y* = (3.93 × 10^−4^ ± 3.81 × 10^−5^)*x* + (3.89 × 10^−5^± 2.71 × 10^−5^) (*r* = 0.997).

### 2.3. Recovery and Matrix Effect

The recovery and matrix effect results are summarized in Table 1. The recoveries of PD were all above 85%, with RSDs lower than 15%. The mean recovery of IS was92.5% with RSD of 3.67%. These data indicated that the recoveries of PD from rat plasma were concentration-independent in the concentration range evaluated and the recoveries were in agreement with international guidelines [15,16], which require that the recovery of each analyte need not be 100%, but the extent of recovery of an analyte and of the internal standard should be consistent, precise, and reproducible. A mean percentage matrix effect value of 93.0% for PD was calculated and found to be independent of PD plasma concentration and rat plasma lot. This result is also in agreement with international guidelines [15,16], and indicates low ion suppression.

**Table 1:** Matrix effects and recoveries of PD in rat plasma (*n* = 5).

Spiked Concentration (ng/mL)	Matrix Effect (%)	Mean ± SD (%)	Recovery (%)	RSD (%)
0.20	93.1	93.0 ± 1.25	88.3	3.27
10.0	94.2		92.0	0.58
160	91.7		90.4	1.44

In the period of method development, the following two pretreatment methods were firstly investigated: liquid-liquid extraction and protein precipitation. Organic solvents are usually necessary in liquid-liquid extraction method and relatively more complicated procedures are also required than those in protein precipitation. However, protein precipitation provides a simple method of sample preparation and has been widely used for the analysis of analytes in plasma. Methanol-acetonitrile (50:50, v/v) was chosen to be the protein precipitator.

### 2.4. Accuracy and Precision of the Assay

To determine the intra-day precision of the method, three plasma samples with the concentrations of 0.20, 10 and 160 ng/mL were analyzed six times on the same day. To determine the inter-day precision and the accuracy, further three plasma samples were run on each of three different days. Table 2 summarizes the intra- and inter-day precision and accuracy for PD from QC samples in rats, respectively. According to the international guidelines [15,16], the mean values of precision and accuracy should be within ±15% of the theoretical values, except at LLOQ, where it should not deviate by more than ±20%.

**Table 2:** Precision and accuracy of the assay method for PD in rat plasma.

Batch	No.	Low	Medium	High
		0.20 ng/mL	10.0 ng/mL	160 ng/mL
Day 1	Mean ± SD	0.20 ± 0.02	10.1 ± 0.46	159 ± 9.04
	RSD (%)	7.75	4.61	5.68
	Accuracy (%)	100.0	100.7	99.5
Day 2	Mean ± SD	0.19 ± 0.01	10.3 ± 0.69	165 ± 7.04
	RSD (%)	7.44	6.64	4.27
	Accuracy (%)	95.0	103.2	103.1
Day 3	Mean ± SD	0.20 ± 0.02	10.2 ± 0.73	163 ± 6.34
	RSD (%)	9.59	7.14	3.88
	Accuracy (%)	97.5	102.0	102.0
Inter-Day	Mean ± SD	0.20 ± 0.02	10.2 ± 0.61	162 ± 7.53
	RSD (%)	8.11	5.95	4.64
	Accuracy (%)	97.5	102.0	101.5

The precision around the mean value should not exceed 15% of the CV, except for LLOQ, where it should not exceed 20% of the CV. All the results indicated that the developed LC-MS/MS method possessed good precision and accuracy.

### 2.5. Stability

The described stability data are summarized in Table 3. The results indicated that PD at the three concentrations tested had acceptable stabilities after three freeze-thaw cycles, at room temperature for 24 h and at −80 °C for 1 month with the % RE values being within ±15%.

### 2.6. Application of the Assay Method

**Figure 4:**
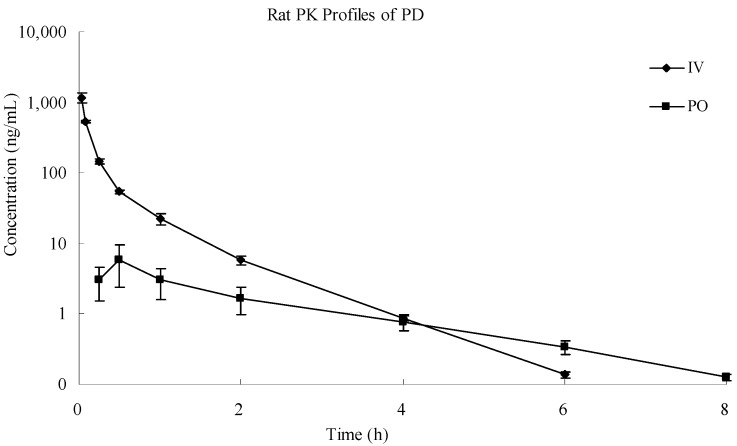
Mean plasma concentration-time profiles of PD determined by LC-MS/MS method after intravenousand oral administration to rats. Each point represents mean ± SD (*n* = 3).

The analytical procedures described were used to quantify PD in the plasma samples obtained from the male SD rats which were intravenously (2.0 mg/kg) and orally (10 mg/kg) administered a single dose of PD. The plasma concentration-time profiles of PD in rats are shown in Figure 4 and the main pharmacokinetic parameters of PD after intravenous and oral administration are presented in Table 4.

**Table 3:** Stability of PD in rat plasma (*n* = 5).

Stability Conditions	Added Conc.	0.20 ng/mL	10.0 ng/mL	160 ng/mL
Three freeze–thaw cycles	Mean ± SD	0.21 ± 0.01	9.89 ± 0.45	161 ± 6.11
	RSD (%)	4.76	4.54	3.80
	Recovery (%)	105.0	98.9	100.4
Room temperature for 24 h	Mean ± SD	0.19 ± 0.02	10.5 ± 0.25	168 ± 4.73
	RSD (%)	7.90	2.39	2.81
	Recovery (%)	96.7	105.3	105.2
Storage at −80 °C for 1 month	Mean ± SD	0.19 ± 0.02	9.66 ± 0.08	151 ± 6.24
	RSD (%)	7.90	0.85	4.14
	Recovery (%)	96.7	96.6	94.4

**Table 4:** Main pharmacokinetic parameters of PD in rats determined after intravenous and oral administration (*n* = 3, mean ± SD).

Parameters	Unit	IV	PO
AUC_(0-t)_	ng/mL × h	185 ± 11.7	10.1 ± 4.28
AUC_(0-∞)_	ng/mL × h	185 ± 11.7	10.4 ± 4.28
MRT_(0-t)_	h	0.36 ± 0.01	2.04 ± 0.32
MRT_(0-∞)_	h	0.36 ± 0.01	2.31 ± 0.43
t_1/2z_	h	0.69 ± 0.06	1.63 ± 0.19
T_max_	h	0.03 ± 0.00	0.50 ± 0.00
CL_z_	L/h/kg	10.8 ± 0.70	1080 ± 436
V_z_	L/kg	10.8 ± 0.96	2626 ± 1340
C_max_	ng/mL	1154 ± 183	5.92 ± 3.51
F	%	-	1.12 ± 0.46

Notes: “t” is the last time point with measurable drug concentration after PO/IV dosing. In the case of PO administration, t equals to 8 h, while in the case of IV administration, t equals to 6 h.

Since PD is insoluble in water, a mixed solvent of DMSO:30% HPCD (5:95, v/v) was adopted in order to obtain a clear solution for intravenous injection. Using this vehicle, a clear PD solution of 2.0 mg/mL was achieved, which was sufficient to meet the requirements of this pharmacokinetic study.

In present study, it was found that PD was rapidly absorbed into the circulation system after oral administration and reached its peak concentration at 30 min. However, its absolute bioavailability was quite low, with a value of about 1.12%. Strong first pass effects and poor permeability through the intestinal epithelial membrane after oral administration might be responsible for the low bioavailability of this compound. Our results will support future pharmacological studies following intravenous administration.

## 3. Experimental Section

### 3.1. Chemicals and Reagents

PD was isolated from the dried plants of *C. longgangensis* var. *hongyao* in our laboratory according to the published method [3], and its purity was determined to be 98.3% by HPLC with photodiode array detection at 332 nm. Genistein (internal standard, IS, purity > 99.0%) was a gift from Shenyang Pharmaceutical University (Shenyang, Liaoning, China). Hydroxypropyl-β-cyclodextrin (HPCD, batch No. 20130928) was purchased from Beijing Fengli Jingqiu Commerce and Trade Co., Ltd (Beijing, China). DMSO of analytical grade was purchased from TEDIA (Cincinnati, OH, USA). Methanol and acetonitrile of HPLC grade were obtained from Fisher Co. Ltd. (Emerson, IA, USA). Formic acid and other reagents were of analytical grade and purchased from MREDA Chemical Reagent Company (Beijing, China). Ultrapure water was produced by a Milli-Q Reagent Water System (Millipore, Billerica, MA, USA).

### 3.2. LC–MS/MS

The HPLC system consisted of an LC-20AD pump, a DGU-20 A_3_ degasser, an SIL-20AC autosampler and a CTO-20A column oven (Shimadzu, Kyoto, Japan). Chromatographic separation was achieved on an Agilent Zorbax XDB C_18_ column (2.1 mm × 50 mm, 3.5 μm), and gradient elution mode was utilized mobile phases consisting of 0.1% formic acid aqueous solution (A) and acetonitrile with 0.1% formic acid (B) with following gradient: 0.00 min (A 80%–B 20%)–0.50 min (A 80%–B 20%)–0.80 min (A 2%–B 98%)–2.00 min (A 2%–B 98%)–2.01 min (A 80%–B 20%)–3.50 min (A 80%–B 20%), with the flow rate of 0.50 mL/min. The injection volume was 10 μL.

The column effluent was monitored using a 4000 QTRAP^®^LC/MS/MS (AB Sciex, Toronto, ON, Canada). The ESI source was operated in negative mode with the curtain, nebulizer and turbo-gas (all nitrogen) set at 20, 60 and 55 psi, respectively. The turbo-gas temperature was 550 °C and the ion spray needle voltage was −4200 V. Multiple reaction monitoring (MRM) was utilized for detecting transition ions from a specific precursor ion to product ion for PD ([M-H]^−^*m/z* 639.2 → 160.9) and the internal standard ([M-H]^−^*m/z* 268.9 → 158.9). The collision energy was set at −55 and −40 eV for PD and the internal standard, respectively.

### 3.3. Preparation of Standards and Calibration Curves

Separate stock solutions of PD and IS (1.0 mg/mL) were prepared by dissolving appropriate amount of each reference standard in DMSO, and were refrigerated until used. A series of PD working standard solutions were prepared by diluting appropriate volumes of PD stock standard solution (1.0 mg/mL) with methanol to obtain the following PD concentrations: 2000, 1000, 500, 200, 100, 50, 20, 10, 5.0, 2.0 and 1.0 ng/mL. These standard solutions of PD were used to spike blank rat plasma with PD to yield calibration standards in plasma over the concentration range of 0.10–200 ng/mL. Briefly, the PD spiking procedure involved transferring a 5 μL aliquot of various PD working standard solutions and a 50 μL aliquot of blank plasma into 1.1 mL centrifuge tubes. Quality control (QC) samples were separately prepared from different weighing of reference standard in same manner at low, medium and high PD levels (0.20, 10, 160 ng/mL). The IS working solutions (100 ng/mL) was prepared by diluting genistein with methanol-acetonitrile (50:50, v/v). All the stock and working standard solutions were stored at 4 °C until analysis.

### 3.4. Sample Preparation

After thawing at room temperature for about 30 min and vortexing for 30 s, aliquots of 50 μL plasma were mixed with 5 μL of methanol (or standard or QC solution) and 150 μL of IS solution (100 ng/mL genistein in methanol-acetonitrile (50:50, v/v)). After vortexing for 1 min and then centrifugation at 12,000 *g* for 10 min, 100 μL aliquots of the supernatants were transferred to HPLC vials. A volume of 10 μL of this solution was then injected onto the column.

### 3.5. Assay Validation

#### 3.5.1. Linearity, Accuracy, Precision, and Recovery

Linear calibration curves in rat plasma were generated by plotting the peak area ratio of PD to the IS *versus* the known plasma PD concentrations over the range of 0.10–200 ng/mL. Slope, intercept and coefficient of correlation were estimated using least squares regression analysis. Quality control samples containing low, medium, and high PD concentrations were used to evaluate the precision and accuracy of the assay method. The intra-day assay precision and accuracy were obtained by analyzing six replicates of the quality controls in duplicate using a calibration curve constructed on the same working day. The inter-day assay precision and accuracy were obtained by analyzing six quality controls in duplicate using calibration curves constructed on three different working days. The precision was indicated by the relative standard deviation (RSD %) and the accuracy was indicated by the relative percentage error from the theoretical concentrations. The lower limit of quantification (LLOQ) was selected as the lowest PD plasma level on the calibration curve. The extraction recoveries of PD from rat plasma (expressed as a percentage) were calculated as the ratios of anun-spiked sample to a post spiked one (a blank sample that has undergone the procedure of sample pretreatment and then is spiked to the corresponding levels).

#### 3.5.2. Stability

The stability of PD in rat plasma was investigated at three QC levels, as described in Section 2.3. Stability tests of the analyte were performed on six replicates of three QC concentrations after: (a) three freeze (−20 °C) and thaw cycles; (b) reconstituted extract at room temperature for 24 h; (c) stored at −80 °C for a month, respectively.

#### 3.5.3. Matrix Effects

Matrix effects from endogenous substances present in extracted rat plasma may cause ion suppression or enhancement of the signal. Matrix effects were assessed by comparing the peak areas of PD after addition of low, medium and high (*n* = 3) concentrations of PD to (A) mobile phase and (B) the supernatant of extracted blank plasma. These studies were conducted with six different lots of rat plasma. The peak area ratio of B/A (as a percentage) or the percentage matrix factor was used as a quantitative measure of the matrix effect.

#### 3.6. Pharmacokinetic Study

The protocols of this animal study were approved by Animal Care and Use Committee of Capital Medical University (Beijing, China). Six male SD rats weighing 200 to 250 g were purchased from Beijing Vital River Laboratories Co., Ltd. (Beijing, China). The rats were certified and had not been dosed with any pharmaceutical before the experiment. The rats were raised under standard laboratory conditions and had *ad libitum* access to water and a standard laboratory diet. Polyethylene cannulas were implanted in the femoral vein 2 days before the experiment after the rats were anesthetized with pentobarbital (50 mg/kg, intravenous). The cannulas were externalized at the back of the neck and filled with heparinized saline (20 units /mL) to prevent blood clotting.

Each rat was housed individually in a rat metabolic cage and was not restrained at any time during the study. The rats were fasted for 16 h before experiments with the exception of free access to water. The dosing solution with PD concentration of 2.0 mg/mL was prepared by dissolving appropriate amount of PD in DMSO-30% HPCD (5:95, v/v). The actual intravenous and oral doses of PD were 2.0 mg/kg and 10 mg/kg, respectively, which were consistent with the medium dose level used in the pharmacological experiments and the dose volumes were both 5.0 mL/kg. After intravenous administration of 2.0 mg/kg PD through tail vein, aliquots of 0.20 mL blood samples were collected in heparinized polyethylene tubes at different time intervals post-dosing (0.033, 0.083, 0.25, 0.50, 1.0, 2.0, 4.0, 6.0 and 8.0 h). After oral administration, aliquots of 0.20 mL blood samples were collected in heparinized polyethylene tubes at different time intervals post-dosing (0.25, 0.50, 1.0, 2.0, 4.0, 6.0 and 8.0 h). Heparinized blood was centrifuged at 12,000 *g* at room temperature for 5 min to obtain plasma which was stored at −80 °C until analysis. Pharmacokinetic parameters including half-life (t_1/2_), maximum plasma time (t_max_) and concentration (C_max_), area under concentration–time curve (AUC_0-t_ and AUC_0-∞_), clearance (CL), steady-state volume of distribution (V_Z_), and mean residence time (MRT) of PD were analyzed by non-compartmental method using DAS Version 2.0 (Chinese Pharmacological Society, Beijing, China). All results were expressed as arithmetic mean ± standard deviation (SD).

## 4. Conclusions

In conclusion, the developed LC-MS/MS method for the determination of PD in rat plasma offers sufficient selectivity, accuracy and precision. The method has been successfully applied to the pharmacokinetic evaluation of intravenous and oral administration of PD using the rat as an animal model, and is currently being applied for further pharmacokinetic characterizations of PD in dogs and monkeys.
